# Prevalence of and factors associated with lactational mastitis in eastern and southern Africa: an exploratory analysis of community-based household surveys

**DOI:** 10.1186/s13006-022-00464-x

**Published:** 2022-03-28

**Authors:** Mariame O. Ouedraogo, Lenka Benova, Tom Smekens, Gezahegn G. Sinke, Abraha Hailu, Herbert B. Wanyonyi, Madalitso Tolani, Caristus Zumbe, Ibukun-Oluwa O. Abejirinde

**Affiliations:** 1 Centre for Global Child Health, The Hospital for Sick Children, Toronto, Canada; 2 Dalla Lana School of Public Health, University of Toronto, Toronto, Canada; 3 Department of Public Health, Institute of Tropical Medicine Antwerp, Antwerp, Belgium; 4 Children Believe, Addis Ababa, Ethiopia; 5 Amref Health Africa Kenya, Nairobi, Kenya; 6 Amref Health Africa Malawi, Lilongwe, Malawi; 7 Amref Health Africa Tanzania, Dar Es Salam, Tanzania

**Keywords:** Lactational mastitis, Sub-Saharan Africa, Prevalence, Associated factors, Exploratory analysis, Maternal morbidity, Newborn health

## Abstract

**Background:**

Lactational mastitis is an extremely painful and distressing inflammation of the breast, which can seriously disrupt breastfeeding. Most of the evidence on the frequency of this condition and its risk factors is from high-income countries. Thus, there is a crucial need for more information on lactational mastitis and its associated factors in Sub-Saharan Africa (SSA).

**Methods:**

We used data from representative, community-based cross-sectional household surveys conducted in 2020 with 3,315 women from four countries (Ethiopia, Kenya, Malawi, and Tanzania) who reported ever-breastfeeding their last child born in the two years before the survey. Our measure of lactational mastitis was self-reported and defined using a combination of breast symptoms (breast redness and swelling) and flu-like symptoms (fever and chills) experienced during the breastfeeding period. We first estimated country-specific and pooled prevalence of self-reported lactational mastitis and examined mastitis-related breastfeeding discontinuation. Additionally, we examined factors associated with reporting mastitis in the pooled sample using bivariate and multivariable logistic regression accounting for clustering at the country level and post-stratification weights.

**Results:**

The prevalence of self-reported lactational mastitis ranged from 3.1% in Ethiopia to 12.0% in Kenya. Close to 17.0% of women who experienced mastitis stopped breastfeeding because of mastitis. The adjusted odds of self-reported lactational mastitis were approximately two-fold higher among women who completed at least some primary school compared to women who had no formal education. Study participants who delivered by caesarean section had 1.46 times higher odds of reporting lactational mastitis than women with a vaginal birth. Despite wide confidence intervals, our models also indicate that young women (15 – 24 years) and women who practiced prelacteal feeding had higher odds of experiencing lactational mastitis than older women (25 + years) and women who did not give prelacteal feed to their newborns.

**Conclusions:**

The prevalence of lactational mastitis in four countries of SSA might be somewhat lower than estimates reported from other settings. Further studies should explore the risk and protective factors for lactational mastitis in SSA contexts and address its negative consequences on breastfeeding.

**Supplementary Information:**

The online version contains supplementary material available at 10.1186/s13006-022-00464-x.

## Background

Mastitis is an inflammatory condition of the breast, commonly associated with lactation, hence the name lactational mastitis [1]. Definitions of lactational mastitis vary somewhat across the literature, however, generally, they combine breast symptoms (breast redness and swelling) and flu-like symptoms (fever and chills) experienced during the breastfeeding period [2]. Lactational mastitis is an extremely painful and distressing condition, estimated to occur in up to 20% of breastfeeding women, and is most frequently reported to occur in the first month of the postpartum period [2].

Most of the evidence on the occurrence of lactational mastitis has emerged from high- and upper-middle-income countries; the three high-quality studies included in a recent meta-analysis of incidence were conducted in China, Iran, and Australia [2]. This meta-analysis estimated that around a quarter of women breastfeeding up to six months of age develop lactational mastitis [2]. In contrast, high-quality data on mastitis from low-income countries, particularly Sub-Saharan Africa (SSA), remains limited. To date, studies conducted in SSA indicate a risk of lactational mastitis ranging between 0.5% and 2.6% [2, 3]. In contrast, results from a relatively large nonrandomized intervention cohort in South Africa suggest that the experience of other breast problems such as blocked ducts and breast engorgement is approximately 15.0% [3].

In addition to the limited evidence on the occurrence of lactational mastitis in SSA countries, minimal information is available on the risk factors of lactational mastitis. In high-income countries, associations between mastitis and anatomical and breastfeeding factors (nipple damage, attachment difficulties, and poor positioning) and obstetric history (primiparity) were found [2]. A study from South Africa indicated that women who exclusively breastfed were less likely to experience breast health problems, including mastitis, than women who did not exclusively breastfeed their newborn [3]. Education and counseling of mothers about optimal breastfeeding techniques have also been suggested to address poor positioning and attachment and ultimately mastitis [4]. Evidence surrounding the importance of sociodemographic factors in experiencing lactational mastitis has been heterogeneous [1].

Recognizing the factors associated with mastitis is important because mastitis can hinder optimal breastfeeding practices, encourage premature supplementary feeding, and lead to breastfeeding cessation. The implications if mastitis leads to breastfeeding cessation may be particularly concerning in low-income countries where access to potable water is challenging and the ability to purchase infant formula is limited. This can impact the growth and development of infants by increasing their exposure to diarrhea and common childhood illnesses, as well as the risk of health problems later in childhood and adolescence [5–8]. Furthermore, mastitis can negatively affect the maternal benefits of breastfeeding, including positive mental health linked to achieving own breastfeeding goals and lower lifetime incidence of type II diabetes and breast and ovarian cancer [7, 9–11].

Given the limited evidence on mastitis and its risk factors in SSA, and the role that lactational mastitis could play in influencing breastfeeding practices, it is important to study mastitis and its associated risk factors in SSA. For this study, we focus on four SSA countries, namely Ethiopia, Kenya, Malawi, and Tanzania. These countries were part of a project designed to reduce maternal and child mortality in specific districts across the four countries through an integrated approach that focuses on strengthening health systems, reducing the burden of diseases, and improving nutrition. Among its nutrition-related initiatives, the project worked towards improving the timely initiation of breastfeeding and exclusive breastfeeding through community-level awareness-raising sessions and training healthcare workers on nutrition-focused services. Key messages conveyed during project activities centered on the benefits, duration, and importance of breastfeeding as well as on good positioning and attachment practices. Across the countries, the prevalence of exclusive breastfeeding ranged between 51% and 84% in 2020, according to project data. In light of these exclusive breastfeeding rates, there is a need to better understand the behaviours and practices related to breastfeeding and the role that lactational mastitis could play in defining those patterns. However, to our knowledge, no study has examined lactational mastitis in those settings of SSA. Considering this, the scarce evidence on mastitis and its risk factors in SSA, and the impact that mastitis can have on breastfeeding continuation, this study aims to investigate the prevalence of and factors associated with lactational mastitis in specific districts of Ethiopia, Kenya, Malawi, and Tanzania. In addition, the present study also documents the breastfeeding habits of women who experienced lactational mastitis.

## Methods

Using cross-sectional data from population-based household surveys, this study aimed to investigate the prevalence of lactational mastitis across four Eastern and Southern Africa countries and explore factors associated with it.

### Study settings

This study used household-level data collected as part of the endline evaluation of large project aiming to improve maternal, newborn and child health and nutrition in specific zones, counties or districts of Ethiopia, Kenya, Malawi, and Tanzania. In Ethiopia, the project was carried out in the Oromia special zone of the Amhara region. Kenya rolled out the project in Siaya county in the western part of the country. In Malawi, the household surveys took place in Chikwawa in the Southern Region and Ntchisi in the Central Region. Finally, in Tanzania, the project and its associated data collection initiatives were conducted in Geita and Nyang’hwale districts, both located in the Geita Region in the northwestern part of the country.

### Data sources

For this study, we used data from the Canada-Africa Initiative to Address Maternal, Newborn, and Child Mortality (CAIA-MNCM) project endline household surveys conducted between February and July 2020 in the four countries of interest. These cross-sectional surveys aimed to provide data representative of the zones, districts, and counties where the project took place. A total of 6,000 households (Ethiopia: *N* = 1,000; Kenya: *N* = 1,000; Malawi: *N* = 2,000; Tanzania: *N* = 2,000) were targeted for participation.

For the household surveys in all four countries, we adopted a two-stage clustered design similar to that employed by the Demographic and Health Surveys (DHS) [12]. The first stage or primary sampling units were the enumeration areas (EA) from the most recent national population census. A sample from the list of all EAs covering the target population was selected using probability proportional to size selection. In the second stage of sampling, a fixed number of households was randomly selected per sampled EA. The selection of households varied among countries: in Ethiopia, we carried out a household listing exercise with the assistance of village leaders and community health workers. In Kenya and Tanzania, we used the household listings recorded by community health workers since they are expected to update household listings at least once a year. In Malawi, we used a random walk approach.

### Study population

The household surveys targeted, amongst other groups, women of reproductive age (15—49 years). Analysis for this study was restricted to the sample of women of reproductive age who had a live birth in the two years before the survey and reported ever breastfeeding their last child born within the two years preceding the survey. A total of 311 women in Ethiopia, 205 in Kenya, 1,339 in Malawi, and 1,460 in Tanzania were eligible.

### Description of variables

The outcome variable of interest was self-reported lactational mastitis at any point during the breastfeeding period related to the last-born child under two years of age. Lactational mastitis was determined by asking the following sequence of questions: *“At any point since you started breastfeeding (name) or within a month of stopping breastfeeding, did you experience the following: breast tenderness or pain, redness of any part of the breast, breast lump?”* and, if women responded positively, *“While you had those symptoms, did you also experience high temperature, flu-like symptoms?”* These questions were developed based on a set of items commonly used in studies of self-reported mastitis [2]. Women who responded positively to both questions were categorized as having experienced lactational mastitis. In addition to collecting information on women’s self-report of mastitis, we inquired about the frequency of symptoms of mastitis and whether the mastitis-related symptoms resulted in stopping breastfeeding. To women who reported experiencing lactational mastitis, we asked these questions specifically: *“Did you have these symptoms more than once while breastfeeding?”* and *“Did you stop breastfeeding as a result of these symptoms?”.*

The explanatory variables were selected after a thorough review of variables used in past studies, which have been documented in a recent systematic review and meta-analysis [2]. Variables included maternal age at the time of the survey (categorised first into five-year intervals: 15–19, 20–24, 25–29, 30–34, 35–39, 40–44, and 45–49 years, and subsequently categorized into larger intervals: 15–24, 25–35, and 36–49 years), time since birth (< 6 months, 6–24 months), parity at the time of index birth (primiparous, multiparous), highest education level (none, less than primary, completed primary, some secondary, higher than secondary), household wealth index constructed following principal component analysis steps defined by the DHS Program [12] and categorised into reasonably equally-sized quartiles (poorest, poorer, richer, richest) within each country, index birth by caesarean section (yes, no), and counseling and observation of breastfeeding during postnatal care (PNC) of the newborn (immediate, delayed, absent/no).

We also derived two variables to measure breastfeeding within the first six months of life. The first variable, applied to the entire sample, was based on the proportion of participating women who reported their newborns receiving any liquids other than breastmilk within the first three days of life (prelacteal feeding). The second variable measuring exclusive breastfeeding during the first six months of life was only applied to a sub-sample of participants whose last-born child was < 6 months of age at time of the survey. It consisted of measuring the proportion of women who indicated not giving liquids and foods other than breastmilk to their newborn in the 24 h before their interview. Both variables were dichotomous (yes, no).

### Statistical analysis

All data cleaning and preparation were done on SAS/STAT® software, version 9.4 of the SAS system (SAS Institute, Cary, NC, USA). We performed the analyses on RStudio version 1.4.1106 [13].

We conducted an initial descriptive analysis to highlight the characteristics of study participants by country. In Malawi and Tanzania, data collection occurred in two districts (as described above). Given this, we generated weighted descriptive statistics to account for different probabilities of household selection in each district. Weights were not used to calculate descriptive statistics in Ethiopia and Kenya as data collection took place in one zone and one county, respectively. We also examined the distribution of self-reported mastitis symptoms and the prevalence of self-reported mastitis in each study setting. Weighted estimates are provided for Malawi and Tanzania.

To increase our statistical power in analysis of risk factors, we pooled data from the four countries. We calculated post-stratification weights in order to account for the countries' differing population sizes. For each country, we estimated the population fraction and the sample fraction. For the population fraction, we divided each country's population size by the sum of the four countries' populations. For the sample fraction, we divided each country's sample size by the total household survey sample size. The final country-specific post-stratification weights were obtained by dividing the population fraction by the sample fraction. An extra step was taken in Malawi and Tanzania, where we calculated district-specific population and sample fractions. These were subsequently multiplied by the country post-stratification weights.

We examined the variables associated with self-reported lactational mastitis through bivariate and multivariable logistic regression models. No statistical tests were used to determine the inclusion of variables in the final regression models. Statistical significance was not the determining factor for selecting variables for the regression models. Rather, it was guided by past work, expert knowledge, and the availability of explanatory variables from the surveys [14, 15]. All results are presented as odds ratios (ORs) with corresponding 95% confidence intervals (95% CI), summarizing the direct effect of the variables on lactational mastitis, instead of their total effects [16]. All models accounted for post-stratification weights and clustering of observations at the country level. The final multivariable logistic regression models excluded parity and counselling on breastfeeding during newborn postnatal care visits due to collinearity with maternal age and observation of breastfeeding during PNC, respectively.

Given that lactational mastitis is more commonly experienced in the first few months following birth and to limit the risk of recall bias, we conducted additional sensitivity analysis restricting the logistic regression to women whose last-born child was < 6-months old at the time of the survey (see Additional file 1). There was a total of 762 women in this sensitivity analysis (Ethiopia: *N* = 104, Kenya *N* = 47, Malawi: *N* = 232, Tanzania: *N* = 379).

## Results

### Characteristics of the study participants

In all four countries, most study participants were age 20–29 years (Table 1). Ethiopia had a large proportion of participants who did not attend formal schooling (67.5%), while in the other countries, most women attended or completed primary school (54.1% in Kenya, 33.4% in Malawi, and 51.4% in Tanzania). In all countries, the majority of participants had more than one child. The caesarean section (c-section) rate was highest in Kenya (11.2%) and the lowest in Ethiopia (2.9%). In Kenya, Malawi, and Tanzania, 72.2%, 58.1%, and 45.9% of women, respectively, indicated being counseled on breastfeeding during their newborn PNC visit, which occurred in the first 48 h following birth. Similarly, in all countries except Ethiopia, many women reported that the postnatal care provider observed their breastfeeding within the first 48 h following birth.

**Table 1:** Background characteristics of the participating women, by study setting

	**Ethiopia ** ***N*** ** (%)**	**Kenya ** ***N*** ** (%)**	**Malawi** ^**a**^ ***N*** ** (%)**	**Tanzania** ^**a**^ ***N *** **(%)**
**Total sample size**	311	205	1,338	1,460
**Maternal age**				
15–19	11 (3.5)	12 (5.8)	97 (8.1)	109 (6.6)
20–24	83 (26.8)	40 (19.5)	399 (29.2)	467 (31.2)
25–29	89 (28.6)	58 (28.3)	340 (24.3)	385 (27.1)
30–34	69 (22.2)	60 (29.3)	246 (18.5)	261 (18.3)
35–39	52 (16.7)	26 (12.7)	187 (14.4)	140 (10.3)
40–44	6 (1.9)	8 (3.9)	53 (4.2)	75 (5.1)
45–49	1 (0.3)	1 (0.5)	16 (1.3)	23 (1.4)
**Time since last live birth**				
< 6 months	104 (33.4)	47 (22.9)	238 (17.8)	389 (26.6)
6 – 24 months	207 (66.6)	158 (77.1)	1100 (82.2)	1071 (73.4)
**Highest education level**				
Never attended school	210 (67.5)	5 (2.4)	283 (23.4)	410 (29.0)
Some primary	78 (25.1)	39 (19.0)	360 (28.5)	129 (9.9)
Completed primary	20 (6.4)	111 (54.1)	474 (33.4)	773 (51.4)
Some secondary	3 (0.9)	40 (19.5)	217 (14.5)	144 (9.2)
Higher	0 (0)	10 (4.9)	3 (0.25)	4 (0.4)
**Household wealth index quartile**				
Poorest	68 (21.9)	41 (20.1)	397 (29.6)	343 (21.9)
Poorer	117 (37.6)	54 (26.5)	379 (29.5)	373 (22.8)
Richer	34 (10.9)	50 (24.5)	271 (20.4)	360 (25.9)
Richest	92 (29.6)	59 (28.9)	288 (20.5)	384 (29.3)
**Parity** ^**b**^				
Primiparous	66 (23.0)	40 (20.8)	312 (25.4)	280 (19.4)
Multiparous	221 (77.0)	152 (79.2)	809 (74.6)	1063 (80.6)
**Most recent birth by caesarean section** ^**c**^				
Yes	9 (2.9)	23 (11.2)	107 (6.9)	53 (3.1)
No	302 (97.1)	182 (88.8)	1229 (93.1)	1407 (96.9)
**Counselling on breastfeeding during PNC of newborn**				
Yes, immediate (48 h after birth)	74 (23.8)	148 (72.2)	777 (58.1)	670 (45.9)
Yes, delayed (> 48 h after birth)	28 (9.0)	24 (11.7)	376 (28.1)	361 (24.7)
No^d^	209 (67.2)	33 (16.9)	185 (13.8)	429 (29.4)
**Observation of breastfeeding during PNC of newborn**				
Yes, immediate (48 h after birth)	66 (21.2)	150 (73.2)	734 (54.9)	684 (46.8)
Yes, delayed (> 48 h after birth)	28 (9.0)	24 (11.7)	335 (25.0)	351 (24.0)
No^d^	217 (69.8)	31 (15.1)	269 (20.1)	425 (29.1)
**Prelacteal feeding**				
Yes	14 (4.5)	12 (5.8)	12 (0.9)	31 (2.1)
No	297 (95.5)	193 (94.1)	1326 (99.1)	1429 (97.9)

*Abbreviation*: *PNC* postnatal care

^a^Weighted frequencies and percentages accounting for different probabilities of household selection in the districts; ^b^Parity data missing for 24 women in Ethiopia, 13 in Kenya, 137 in Malawi, and 117 in Tanzania; ^c^Caesarean section data missing for three women in Malawi who did not respond; ^d^Includes participants who did not receive counselling on/observation of breastfeeding within their *PNC* and those who did not receive *PNC* at all

### Prevalence of self-reported lactational mastitis and characteristics of women reporting lactational mastitis

The prevalence of self-reported lactational mastitis since the birth of their most recent child ranged from 3.1% in Ethiopia to 12.0% in Kenya (Table 2). Among women with self-reported lactational mastitis, 61.0% indicated having experienced mastitis-related symptoms more than once (30.0% in Ethiopia, 68% in Kenya, 51.4% in Malawi, and 75.1% in Tanzania). In addition, across the four countries, about 17.0% of women with lactational mastitis reported stopping breastfeeding due to mastitis (20.0% in Ethiopia, 20.0% in Kenya, 20.5% in Malawi, and 9.2% in Tanzania).

**Table 2:** Distribution of self-reported lactational mastitis symptoms and estimated prevalence of self-reported lactational mastitis among the study participants, by country and pooled (*N* = 3,314)

	**Ethiopia ** ***N*** ** (%)**	**Kenya ** ***N*** ** (%)**	**Malawi** ^**a**^ ***N*** ** (%)**	**Tanzania** ^**a**^ ***N*** ** (%)**	**Pooled ** ***N*** ** (%)**
**Total sample size**	311	205	1,338	1,460	3,314
**Mastitis symptoms**					
Breast tenderness or pain	10 (3.1)	54 (25.9)	149 (11.0)	207 (14.0)	382 (11.5)
Redness of any part of the breast	10 (3.1)	24 (11.5)	50 (3.7)	80 (5.4)	186 (5.6)
Breast lump	13 (4.1)	32 (15.4)	50 (3.7)	89 (6.0)	237 (7.1)
**In addition to at least one of the symptoms above**					
Fever	9 (60.0)	21 (35.6)	56 (32.0)	94 (39.3)	183 (41.3)
Flu-like symptoms	7 (46.7)	24 (39.3)	59 (33.7)	106 (44.3)	189 (41.8)
**Prevalence of mastitis**					
	10 (3.1)	25 (12.0)	73 (5.4)	124 (8.4)	222 (6.7)

^a^Weighted frequencies and percentages accounting for different probabilities of household selection in the districts

As shown for the pooled sample in Table 3, self-reported lactational mastitis was most commonly observed among participants aged between 15 and 24 years (8.0%). About 7.3% of women who were 6–24 months postpartum reported lactational mastitis, compared to 5.2% of women who were within the first six months of the postpartum period. Close to 4.0% of women who never attended formal education experienced lactational mastitis, while 9.3% of respondents part of the poorest wealth quantile indicated mastitis. We found no evidence of important differences in the proportion of self-reported mastitis by parity nor by counselling and observation of breastfeeding during postnatal care. We also observed that 11.3% of participating women who gave birth by c-section had self-reported lactational mastitis, compared to 6.5% of women who had a vaginal delivery. In addition, reporting of lactational mastitis was higher among women who reported their newborns receiving prelacteal feed (13.7%) compared to 6.4% among women who did not. Similar bivariate associations between explanatory factors and experience of lactational mastitis were found in the sub-group analysis of women < 6 months postpartum (see Additional file 1, Table A1).

**Table 3:** Reporting of lactational mastitis by women according to selected explanatory factors, pooled sample (*N* = 222 women with self-reported lactational mastitis)

	**Weighted frequency of women reporting lactational mastitis**	**Weighted % of women reporting lactational mastitis**	***P*** **-value**
**Maternal age** ^**a**^			
15 – 24 years	84	8.0	0.1418
25 – 35 years	117	6.4	
36 – 49 years	21	4.7	
**Time since birth**			
< 6 months	49	5.2	< 0.0001
6–24 months	173	7.3	
**Highest education level**			
Never attended school	52	3.9	0.0045
Some primary	56	8.3	
Completed primary	86	8.8	
Some secondary or higher	28	9.1	
**Household wealth index quartile**			
Poorest	68	9.3	0.4062
Poorer	62	6.0	
Richer	49	8.1	
Richest	43	4.6	
**Parity**			
Primiparous	58	7.9	0.4007
Multiparous	162	6.8	
**Most recent birth by caesarean section**			
Yes	19	11.3	0.0845
No	203	6.5	
**Counselling on breastfeeding during PNC of newborn**			
Yes, immediate (48 h after birth)	101	7.2	0.9306
Yes, delayed (> 48 h after birth)	32	6.5	
No^b^	89	6.3	
**Observation of breastfeeding during PNC of newborn**			
Yes, immediate (48 h after birth)	104	7.5	0.7010
Yes, delayed (> 48 h after birth)	31	6.5	
No^b^	87	6.0	
**Prelacteal feed**			
Yes	18	13.7	0.1181
No	204	6.4	

*Abbreviation*: *PNC* postnatal care

^a^ Maternal age was collapsed into broader categories due to sample size limitations; ^b^Includes participants who did not receive counselling on/observation of breastfeeding within *PNC* and those who did not receive *PNC* at all

###  Logistic regression analyses of factors associated with self-reported lactational mastitis

The bivariate logistic regression analyses (Table 4) indicated that being 25—35 years was associated with lower odds of reporting lactational mastitis compared to being aged between 15 and 24 years. On the other hand, having at least some primary education was associated with higher odds of self-reported lactational mastitis, with the stronger association being reported when comparing women who never attended school with their counterparts who attended at least some secondary school (unadjusted OR 2.74; 95% CI 1.19, 6.31). Although not statistically significant, women who were primiparous had 1.17 times higher odds of self-reported lactational mastitis than multiparous women. Women who gave birth by c-section were 84% more likely to self-report experiencing mastitis than women who delivered vaginally. Prelacteal feeding was associated with increased odds of self-reported lactational mastitis. The confidence interval was however large, suggesting some variability and thus moderate evidence that women who practice prelacteal feeding have higher likelihood of experiencing lactational mastitis.

**Table 4:** Bivariate and multivariable logistic regression of selected explanatory factors and lactational mastitis, pooled sample (*N* = 3,314)

	**Unadjusted odds ratio (95% CI)**	**Adjusted odds ratio (95% CI)**
**Age** ^**a**^		
15 – 24 years	1.0	1.0
25 – 35 years	0.79 (0.64, 0.96)	0.88 (0.68, 1.13)
36 – 49 years	0.56 (0.24, 1.31)	0.71 (0.33, 1.54)
**Time since birth**		
< 6 months	1.0	1.0
6–24 months	1.42 (1.38, 1.47)	1.46 (1.37, 1.55)
**Highest education level**		
Never attended school	1.0	1.0
Some primary	2.21 (1.24, 3.95)	2.19 (1.18, 4.06)
Completed primary	2.38 (1.01, 5.60)	2.26 (0.96, 5.29)
Some secondary or higher	2.46 (1.17, 5.19)	2.74 (1.19, 6.31)
**Household wealth index quartile**		
Poorest	1.0	1.0
Poorer	0.63 (0.26, 1.51)	0.65 (0.28, 1.49)
Richer	0.86 (0.79, 0.93)	0.75 (0.66, 0.85)
Richest	0.47 (0.15, 1.52)	0.42 (0.13, 1.35)
**Parity**		
Multiparous	1.0	^b^
Primiparous	1.17 (0.81, 1.69)	
**Most recent birth by caesarean section**		
No	1.0	1.0
Yes	1.84 (0.91, 3.74)	1.46 (1.07, 2.00)
**Counselling on breastfeeding during PNC of newborn**		
Yes, immediate (48 h after birth)	1.0	^b^
Yes, delayed (> 48 h after birth)	1.15 (0.41, 3.21)	
No^c^	1.04 (0.65, 1.68)	
**Observation of breastfeeding during PNC of newborn**		
Yes, immediate (48 h after birth)	1.0	1.0
Yes, delayed (> 48 h after birth)	1.28 (0.57, 2.88)	1.07 (0.38, 3.03)
No^c^	1.10 (0.76, 1.58)	0.99 (0.53, 1.89)
**Prelacteal feed**		
No	1.0	1.0
Yes	2.31 (0.78, 6.82)	2.15 (0.70, 6.61)

*Abbreviations*: *CI* confidence interval, *PNC* postnatal care

^a^Maternal age was collapsed into broader categories due to sample size limitations; ^b^Excluded from the final model due to collinearity; ^c^Includes participants who did not receive counselling on/observation of breastfeeding within *PNC* and those who did not receive *PNC* at all

The multivariable logistic regression models (Table 4) show that the odds of lactational mastitis were 46% greater in women 6–24 months postpartum than in women who were in the first five months of the postpartum phase. Additionally, the odds of self-reported mastitis were approximately two-fold higher among women who completed at least some primary school. Birth by c-section was significantly associated with higher odds of self-reported lactational mastitis (adjusted OR 1.46; 95% CI 1.07, 2.00). Despite not being statistically significant, the models also suggest that younger maternal age and giving prelacteal feed were associated with higher likelihood of lactational mastitis.

Even though wide confidence intervals were noted due to small sample size, the direction of the ORs between the considered explanatory variables and self-reported lactational mastitis were also confirmed in our sub-group analyses of women whose last-born child was < 6 months at the time of the survey. In these analyses, we observed weak evidence that women who did not exclusively breastfeed their newborns had higher odds of lactational mastitis than women who exclusively breastfed their newborns (adjusted OR 2.55; 95% CI 0.66, 9.80, Additional file 1).

## Discussion

In this study, we estimated the prevalence of lactational mastitis and its associated factors in representative samples of breastfeeding women in Ethiopia, Kenya, Malawi, and Tanzania. It aimed to address the dearth of evidence on lactational mastitis in low- and middle- income countries, particularly in SSA.

Analysis showed a pooled prevalence of mastitis among breastfeeding women of 6.7%, with the lowest prevalence noted in Ethiopia (3.1%) and the highest in Kenya (12.0%). More than half of women who experienced mastitis reported multiple episodes. Our estimates are somewhat lower than those reported in previous studies conducted in other settings. For instance, a prospective cohort study of nulliparous women in Australia reported an incidence of lactational mastitis of 20.0% [17]. Another small cohort study conducted in Iran found that about 19.0% of women experienced mastitis [18]. In contrast, in China, a lower proportion of breastfeeding women reported experiencing at least one episode of mastitis (6.3%) [19]. Our prevalence of mastitis is somewhat close to the incidence detected in a US-based cohort study of 9.5% [20]. Our pooled prevalence estimate of lactational mastitis is also higher than incidences noted in HIV-infected and uninfected women in South Africa (1.0% and 0.5%, respectively) [3], but closer to what has been observed in Nepal (8.0%) [21]. The prevalence we identified in Kenya is similar to that noted in a cross-sectional assessment of Ghanaian women at a breast care center (11.8%) [22].

Several reasons can explain the differences between our findings and those observed in other countries. First, there are considerable differences in the definition and measurement of mastitis across studies. For instance, some studies have considered diagnosis by healthcare providers [19] or the incidence of breast and systemic symptoms within a specific time frame [17], while our study was based on self-reported symptoms of mastitis during up to two years preceding participation in the household surveys. A recent systematic review and meta-analysis demonstrated substantial variability in the definitions of mastitis used in studies, but also in the population of focus (general population versus hospital-based population) [2]. Additionally, prospective cohort studies can provide more accurate measures of mastitis incidence. As such, our study, which estimated self-reported prevalence retrospectively, may be underestimating the true frequency of lactational mastitis in the four countries. Finally, differences between the countries considered in this study and other settings is likely linked to differences in breastfeeding practices [2]. Another hypothesis explaining differences in mastitis prevalence pertains to variabilities in maternal gut bacteria, leading to distinct milk microbiome in the different countries [2, 23].

An important result of our analysis is that 17.0% of women who indicated experiencing mastitis stopped breastfeeding their infants because of mastitis-related symptoms. Our analysis was however limited in its ability to detect the distinct sociodemographic profiles of these women. Continued breastfeeding despite mastitis symptoms has been noted in a qualitative study conducted in Burkina-Faso. In that study, breastfeeding was reported by the participants to be one of the most effective way to alleviate symptoms and prevent the development of other breast complications [24]. Another explanation reported in the same article is that the lack of access to affordable and safe alternatives to breast milk may result in women preferring or being advised to continue breastfeeding [24]. A quantitative study from New Zealand also found a positive association between experiencing mastitis and breastfeeding duration [25]. Further quantitative and qualitative studies into the beliefs, attitudes, and practices of women experiencing mastitis and other clinical issues related to mastitis will be important to understand the enabling factors supporting continued breastfeeding.

Breastfeeding women who had some education and delivered by c-section had higher odds of reporting experiencing mastitis. These results mirror those found in other studies [21, 26]. Women who have some education may be working in formal settings preventing them from establishing and maintaining regular feeding practices to decrease the risk of milk stasis. There is also a possibility that women with higher levels of education were more likely to have understood the survey questions and therefore more likely to report experiencing mastitis symptoms.

The link between c-section and mastitis observed in this study is consistent with findings of a prospective cohort study conducted in Nepal which reported that women who gave birth by c-section were at higher risk of mastitis [21]. A potential explanation raised by the authors is that c-section is associated with delayed initiation of breastfeeding and therefore increased risk of milk stasis, a major risk factor for mastitis. Another study identified that, compared to women who had a vaginal birth, women who delivered by c-section had a higher proportion of breastfeeding difficulties [27]. In contrast, a case–control study with breastfeeding women from Australia with and without mastitis did not find an association between method of birth and mastitis [28].

The relationship of mastitis and household wealth quantile is interesting to note, as we expected wealth and education to be highly correlated and thus related to lactational mastitis in a similar way. Very few studies have looked at changes in the frequency of mastitis by a measure of socioeconomic status (SES), such as household wealth. However, Vogel et al. found that high SES was associated with higher odds of reported mastitis (OR 1.13) [25], which is in accordance with the relationship we observed between education and mastitis. Similarly, a study from Australia highlighted that women from wealthier households and with private health insurance had lower odds of lactational mastitis [28]. There are possible pathways through which high SES could lead to a reduced likelihood of lactational mastitis. For instance, compared to women from poorer households, those from richer households may have greater access to health services and supportive counseling on breastfeeding and information to prevent mastitis. An alternative explanation is that women from wealthier households may have access to water for handwashing, thus limiting the transfer of bacteria to the breast during the breastfeeding process. Future studies are needed to elucidate the pathways through which SES could influence the risk of lactational mastitis.

While the confidence intervals for the ORs for the relationships of maternal age and prelacteal feeding practices with mastitis were wide in our study, the direction of the ORs suggests that they may be important factors associated with mastitis. The current state of the literature provides inconsistent insights into the association between maternal age and lactational mastitis [2]. In addition, very few studies have examined the association between prelacteal feeding and mastitis. Still, a cohort study from Nepal supports the hypothesis that the risk of lactational mastitis is higher in women who practice prelacteal feedings than in their counterparts who did not engage in prelacteal feeding [21]. Prelacteal feeding, defined as introducing foods and liquids other than breastmilk to newborns in the first few days of life [29], could lead to milk stasis, a major risk factor for mastitis [21]. Future studies with larger sample sizes should investigate the potential associations of maternal age and prelacteal feeding.

### Strengths and limitations

The main strength of this study is that we used large representative household surveys across four SSA countries to address the scarcity of information on the prevalence of lactational mastitis and related breastfeeding discontinuation in these settings. In the four countries, we had high participation rates (more than 95% in each country), thus limiting the risks of non-response bias.

Still, there are important limitations to be considered. First, the associations are based on cross-sectional data; we, therefore, cannot interpret any of the relationships presented here as causal. Another limitation is that all the variables included in this study were based on self-report, hence increasing the chances of recall bias and misclassification. Although we do not think that women are likely to underreport experiencing mastitis, to appraise potential bias in reporting, we compared mastitis among women < 6 months postpartum and those 6–24 months postpartum. We found that the odds of ever experiencing mastitis were higher among women 6–24 months, consistently with the longer time at risk of the condition. We also attempted to overcome the risk of recall bias by restricting our sensitivity analyses to women less than six-month postpartum. Some of the variables considered in this study may have also been underestimated or overestimated because of social desirability bias or challenges understanding the questions. We attempted to ease the comprehension of the questions by administering the surveys in local languages and training the data collectors on the technical interpretation of survey terms. Also, the majority of the questions (except from the mastitis questions) were framed based on standardized DHS questionnaires.

Because of sample size limitations, we could not conduct country-specific logistic regression analyses. Similarly, while the directions of the ORs from the sensitivity analyses were aligned to those reported for the full sample, the sample size limited our ability to get precise ORs. Future studies will be needed to further document the risk factors of lactational mastitis early in the postpartum period. Finally, our surveys did not include information on other key factors such as breast characteristics and history of mastitis. More studies should explore additional variables capturing potential anatomical, sociodemographic, individual and health facility factors related to the occurrence and experience of lactational mastitis.

## Conclusions

In this study, we have for the first time estimated the prevalence of lactational mastitis in four countries of Eastern and Southern Africa. We found that, similarly to a range of estimates from other settings, between three and 12 in 100 women with a recent birth self-reported experiencing the condition, and more than half of them more than once. Across the four countries, we observed variability in the occurrence of mastitis among breastfeeding women. Our study shows that a simple set of questions can be used in surveys to collect prevalence of lactational mastitis retrospectively. Further, it suggests that younger maternal age, higher education, birth by c-section, and prelacteal feeding are associated with higher likelihood of lactational mastitis in these settings. However, further investigations into the occurrence of lactational mastitis and its predisposing factors in Africa, particularly among women up to six-months postpartum, are required to ensure optimal breastfeeding practices. Additionally, more studies on the experiences of women with lactational mastitis are needed, including the factors enabling continued breastfeeding after a mastitis episode and the support and care women prefer and desire, for breastfeeding in general, but also for mastitis in particular.

## Supplementary Information

**Additional file 1.** Sensitivity analyses with breastfeeding women 0–5 months postpartum.

## Data Availability

The datasets used and/or analyzed during this study cannot be made available, as the study participants did not agree to share their data with organizations or research groups outside of those part of the CAIA-MNCM consortium.

## Abbreviations

CAIA-MNCM: Canada-Africa Initiative to Address Maternal, Newborn, and Child Mortality
CI: Confidence Interval
C-section: Caesarean section
DHS: Demographic and Health Survey
EA: Enumeration Area
OR: Odds Ratio
PNC: Postnatal Care
SES: Socio-economic status
SSA: Sub-Saharan Africa
