# Supplementary material for: Prevalence of and factors associated with lactational mastitis in eastern and southern Africa: an exploratory analysis of community-based household surveys
Source: Int Breastfeed J. 2022 Mar 28;17:24. doi: 10.1186/s13006-022-00464-x (PMC8962073; doi:10.1186/s13006-022-00464-x)
Supplement: Supplementary file 1 — Additional file 1. Sensitivity analyses with breastfeeding women 0–5 months postpartum. [file 13006_2022_464_MOESM1_ESM.docx]

**ADDITIONAL FILE 1**

**Prevalence of and Factors Associated with Lactational Mastitis in Eastern and Southern Africa: An Exploratory Analysis of Community-based Household Surveys**

Mariame O. Ouedraogo^1,2*^, Lenka Benova^3^, Tom Smekens^3^, Gezahegn G. Sinke^4^, Abraha Hailu^4^, Herbert B. Wanyonyi^5^, Madalitso Tolani^6^, Caristus Zumbe^7^, Ibukun-Oluwa O. Abejirinde^1,2^

^1^Centre for Global Child Health, The Hospital for Sick Children, Toronto, Canada

^2^Dalla Lana School of Public Health, University of Toronto, Toronto, Canada

^3^Department of Public Health, Institute of Tropical Medicine Antwerp, Antwerp, Belgium

^4^Children Believe, Addis Ababa, Ethiopia

^5^Amref Health Africa Kenya, Nairobi, Kenya

^6^Amref Health Africa Malawi, Lilongwe, Malawi

^7^Amref Health Africa Tanzania, Dar Es Salam, Tanzania

* Corresponding author

E-mail: [mariame.ouedraogo@sickkids.ca](mailto:m.ouedraogo@mail.utoronto.ca) (MOO)

**Table A1. Reporting of lactational mastitis by women 0-5 months postpartum according to selected explanatory factors, pooled data from 4 countries (N=49)**

|  | **Weighted frequency of women reporting lactational mastitis** | **Weighted % of women reporting lactational mastitis** | **P-value** |
| --- | --- | --- | --- |
| **Maternal age^a^** | | | |
| 15 – 24 years | 20 | 5.4 | 0.0573 |
| 25 – 35 years | 27 | 5.8 |  |
| 36 – 49 years | 2 | 1.5 |  |
| **Highest education level** | | | |
| Never attended school | 6 | 1.4 | 0.0701 |
| Some primary | 22 | 11.9 |  |
| Completed primary | 19 | 7.1 |  |
| Some secondary or higher | 2 | 3.5 |  |
| **Household wealth index quartile** | | | |
| Poorest | 15 | 6.1 | 0.6712 |
| Poorer | 17 | 5.7 |  |
| Richer | 11 | 7.1 |  |
| Richest | 6 | 2.6 |  |
| **Parity** | | | |
| Nulliparous | 17 | 7.0 | 0.0319 |
| Multiparous | 32 | 5.0 |  |
| **Most recent birth by caesarean section** | | | |
| Yes | 6 | 22.9 | 0.1223 |
| No | 43 | 4.7 |  |
| **Counselling on breastfeeding during PNC of newborn** | | | |
| Yes, immediate (48 hours after birth) | 16 | 4.6 | 0.5293 |
| Yes, delayed (>48 hours after birth) | 4 | 3.7 |  |
| No^b^ | 29 | 6.1 |  |
| **Observation of breastfeeding during PNC of newborn** | | | |
| Yes, immediate (48 hours after birth) | 20 | 5.8 | 0.6283 |
| Yes, delayed (>48 hours after birth) | 4 | 3.6 |  |
| No^b^ | 25 | 5.2 |  |
| **Exclusive breastfeeding for 0-5 months** | | | |
| Yes | 19 | 9.2 | 0.2167 |
| No | 30 | 3.1 |  |

^a^ Maternal age were collapsed into broader categories due to sample size limitations; ^b^Includes participants who did not receive counselling on/observation of breastfeeding within PNC and those who did not receive PNC at all. Abbreviations: PNC – postnatal care

**Table A2. Univariate and multivariate logistic regression of selected explanatory factors and lactational mastitis among 6-month postpartum women, overall**

|  | **Unadjusted odds ratio (95% CI)** | **Adjusted odds ratio**  **(95% CI)** |
| --- | --- | --- |
| **Maternal age^a^** | | |
| 15 – 24 years | 1.0 | 1.0 |
| 25 – 35 years | 1.08 (0.89, 1.30) | 1.49 (0.52, 4.28) |
| 36 – 49 years | 0.28 (0.06, 1.24) | 0.52 (0.10, 2.68) |
| **Highest education level** | | |
| Never attended school | 1.0 | 1.0 |
| Some primary | 9.61 (0.68, 136.06) | 7.77 (0.85, 71.00) |
| Completed primary | 5.42 (0.30, 96.57) | 5.09 (0.18, 144.20) |
| Some secondary or higher | 2.57 (0.27, 24.03) | 3.0.4 (0.22, 41.62) |
| **Household wealth index quartile** | | |
| Poorest | 1.0 | 1.0 |
| Poorer | 0.92 (0.64, 1.30) | 0.76 (0.36, 1.62) |
| Richer | 1.16 (0.36, 3.74) | 0.91 (0.43, 1.92) |
| Richest | 0.41 (0.05, 3.44) | 0.40 (0.05, 3.10) |
| **Parity** | | |
| Multiparous | 1.0 | ^b^ |
| Nulliparous | 1.43 (1.03, 1.99) |  |
| **Most recent birth by caesarean section** | | |
| No | 1.0 | 1.0 |
| Yes | 5.99 (0.46, 77.60) | 3.58 (0.32, 40.36) |
| **Counselling on breastfeeding during PNC of newborn** | | |
| Yes, immediate (48 hours after birth) | 1.0 | ^b^ |
| Yes, delayed (>48 hours after birth) | 0.75 (0.40, 1.40) |  |
| No^c^ | 0.59 (0.15, 2.37) |  |
| **Observation of breastfeeding during PNC of newborn** | | |
| Yes, immediate (48 hours after birth) | 1.0 | 1.0 |
| Yes, delayed (>48 hours after birth) | 1.11 (0.77, 1.59) | 0.81 (0.20, 3.30) |
| No^c^ | 0.68 (0.17, 2.64) | 0.68 (0.10, 4.40) |
| **Exclusive breastfeeding for 6 months** | | |
| No | 1.0 | 1.0 |
| Yes | 3.15 (0.47, 21.28) | 2.56 (0.68, 9.62) |

^a^ Maternal age were collapsed into broader categories due to sample size limitations^; b^ Excluded from the final model due to collinearity; ^c^Includes participants who did not receive counselling on/observation of breastfeeding within PNC and those who did not receive PNC at all. Abbreviations: CI – confidence interval; PNC – postnatal care
